# The Role of Oligodendrocytes in Neurodegenerative Diseases: Unwrapping the Layers

**DOI:** 10.3390/ijms26104623

**Published:** 2025-05-12

**Authors:** Leona Bokulic Panichi, Stefano Stanca, Cristina Dolciotti, Paolo Bongioanni

**Affiliations:** 1 Neuroscience Department, Azienda Ospedaliero-Universitaria Pisana, 56126 Pisa, Italy; 2 NeuroCare Onlus, 56124 Pisa, Italy; 3 Department of Surgical, Medical, Molecular Pathology and Critical Area, University of Pisa, 56126 Pisa, Italy

**Keywords:** neurodegenerative diseases, oligodendrocytes, oligodendrocyte progenitor cells, myelination, demyelination, remyelination

## Abstract

Neurodegenerative diseases (NDs), including Alzheimer’s disease, Parkinson’s disease, amyotrophic lateral sclerosis/motor neuron disease, and multiple sclerosis, are characterized by progressive loss of neuronal structure and function, leading to severe cognitive, motor, and behavioral impairments. They pose a significant and growing challenge due to their rising prevalence and impact on global health systems. The societal and emotional toll on patients, caregivers, and healthcare infrastructures is considerable. While significant progress has been made in elucidating the pathological hallmarks of these disorders, the underlying cellular and molecular mechanisms remain incompletely understood. Increasing evidence implicates oligodendrocytes and their progenitors—oligodendrocyte progenitor cells (OPCs)—in the pathogenesis of several NDs, beyond their traditionally recognized role in demyelinating conditions such as MS. Oligodendrocytes are essential for axonal myelination, metabolic support, and neural circuit modulation in the central nervous system. Disruptions in oligodendrocyte function and myelin integrity—manifesting as demyelination, hypomyelination, or dysmyelination—have been associated with disease progression in various neurodegenerative contexts. This review consolidates recent findings on the role of OPCs in NDs, explores the concept of myelin plasticity, and discusses therapeutic strategies targeting oligodendrocyte dysfunction. By highlighting emerging research in oligodendrocyte biology, this review aims to provide a short overview of its relevance to neurodegenerative disease progression and potential therapeutic advances.

## 1. Introduction

Neurodegenerative diseases (NDs) encompass a broad class of progressive disorders marked by the deterioration of neuronal structure and function. They are among the most challenging health problems of the 21st century; Alzheimer’s disease (AD), Parkinson’s disease (PD), amyotrophic lateral sclerosis/motor neuron disease (ALS/MND), and multiple sclerosis (MS) collectively affect millions of individuals worldwide, with numbers anticipated to rise significantly by 2050, a trend largely due to an aging population [1]. These conditions present with heterogeneous clinical manifestations—ranging from cognitive impairment in AD to motor dysfunction in PD and ALS, and the multifaceted neurological symptoms of MS—depending on the affected neural circuits and underlying molecular pathology [2].

Beyond their clinical burden, NDs impose a substantial and growing economic and emotional toll on patients, caregivers, and healthcare systems [3,4]. The cost includes direct medical expenses—such as hospitalization, medication, and long-term care—and indirect costs, including reduced productivity and psychological distress. In the United States, the annual cost of dementia care was estimated at USD 305 billion in 2020, projected to exceed USD 1 trillion by 2050. Globally, dementia-related costs reached USD 1 trillion in 2018 and are expected to double by 2030 [5].

Given the clinical heterogeneity and multifactorial nature of NDs, there is a growing need for integrative research frameworks that can account for the diverse neuroanatomical and molecular factors driving disease progression. Despite decades of intensive research, many of the core mechanisms driving neurodegeneration remain incompletely understood, and effective disease-modifying treatments remain elusive, underscoring the need for integrative frameworks that capture the complexity of disease progression across neuroanatomical, cellular, and molecular domains. This includes advancing our understanding of the pathophysiological mechanisms underlying these conditions, developing effective treatments and interventions, and implementing supportive policies for patients and caregivers. The study of NDs remains a critical area of research with significant implications for improving patients’ care and outcomes.

While protein aggregation, synaptic dysfunction, and neuroinflammation have been widely studied, growing evidence suggests that glial cells—including astrocytes, microglia, and oligodendrocytes and their progenitors—play a more active role in disease progression than previously appreciated. Oligodendrocytes and their progenitors, oligodendrocyte progenitor cells (OPCs), are gaining recognition for their contributions to neuronal function and vulnerability in the context of NDs. Oligodendrocytes are essential for axonal myelination and metabolic support in the central nervous system (CNS), and their dysfunction has been increasingly implicated in the pathogenesis of multiple neurodegenerative disorders. For instance, early myelin loss and oligodendrocyte atrophy have been observed in AD and correlate with cognitive decline; in PD, demyelination and α-synuclein accumulation may impair their function; in ALS, the loss of oligodendrocyte support contributes to motor neuron vulnerability; and in MS, primary immune-mediated demyelination directly targets oligodendrocytes. These findings underscore the relevance of oligodendrocyte dysfunction across diverse NDs and suggest novel opportunities for therapeutic intervention.

Table 1 provides an overview of the main symptoms, oligodendrocyte pathology, and potential therapeutic targets in the major neurodegenerative diseases discussed.

This review aims to synthesize current knowledge on the role of OPCs and oligodendrocytes in NDs. It highlights how disruptions in myelin integrity, oligodendrocyte dysfunction, and impaired glial–neuronal communication contribute to disease mechanisms. Special attention is given to the concept of myelin plasticity and its implications for remyelination and neural resilience. By illuminating the multifaceted roles of oligodendrocytes in neural resilience and degeneration, this work aims to chart a path forward for therapeutic innovation in neurodegenerative diseases.

## 2. Myelin: From Its Discovery to Its Dynamic Role in Nervous Transmission and Plasticity

The advent of the myelin sheath represents a pivotal evolutionary advancement in vertebrates, facilitating complex nervous systems and higher-order behaviors. Antonie van Leeuwenhoek, in the early 18th century, is often recognized as the first to notice and report on the myelin sheath, although it took another century and a half for the term “myelin” to be introduced by Rudolf Virchow. In 1858, misinterpreting its location as inside the axon’s empty space and likening it to bone marrow, Virchow named it “myelin” [6], inspired by the Greek for marrow, myelos [μυελός], describing good and pure bodily fluid flowing through a channel connecting the spine and genitals and lower limbs [7].

Following the discovery of Leeuwenhoek in 1717, theories about myelin began to emerge, with significant contributions and debates over the centuries. The understanding of myelin’s origins remained elusive until Ranvier’s interpretation of myelin as the product of a type of fatty cell (1872), a theory initially met with skepticism due to the invisible nature of the cytoplasm [8]. It was only in 1919, through the pioneering histological studies of Pío del Río-Hortega, that oligodendrocytes—the CNS’s myelinating cells—were conclusively identified, gaining wider acceptance through supportive work by Wilder Penfield in 1924 [9,10]. Investigations into myelin’s fatty nature and structure evolved concurrently, with significant advancements in understanding its crystal-like organization and the discovery of saltatory conduction, highlighting the essential function of myelin. The confirmation by Richard and Mary Bunge in 1962, demonstrating oligodendrocytes’ definitive role in CNS myelination, catalyzed modern myelin biology research [11,12].

Today, it is universally accepted that the role of myelin is more than simply increasing the speed of electrical signals traveling through the axons by saltatory conduction, allowing the action potential to be propagated from one unmyelinated node of Ranvier to the other [13]. Previous perceptions of myelin as static and unchangeable have evolved due to findings that myelin undergoes alterations over an individual’s lifetime, influenced by experiences or by learning new skills [14]. These changes adjust how neuronal networks operate, particularly those associated with learning and memory [15]. In addition to facilitating rapid signal transmission, myelin plays a crucial role in preserving axonal integrity, regulating axonal architecture, and supporting metabolic efficiency—particularly in long-projecting neurons, where reduced ATP consumption is essential for maintaining ion homeostasis [16].

Myelin’s composition and architecture are intricate, predominantly lipid-rich but interspersed critically by proteins such as myelin basic protein (MBP) and proteolipid protein (PLP). MBP is vital for myelin’s structural integrity and compaction, functioning as an “executive molecule” regulating membrane interactions [17]. PLP, encoded by the PLP1 gene and expressed in oligodendrocytes and other glial cells [18], not only contributes structurally but mutations therein lead to diverse myelin disorders, notably multiple sclerosis (MS), spastic paraplegia type 2 [19], and Pelizaeus–Merzbacher disease (PMD) [20]. These proteins, combined with specific lipids (cholesterol, galactocerebroside), create a tightly organized lamellar structure, essential for effective electrical insulation and metabolic support to neurons [21].

Considering the importance of myelin, it is imperative to understand the process of *myelinogenesis*, the formation of myelin sheaths, which refers to the developmental period during which myelin is initially produced, highlighting the cellular and molecular mechanisms underlying myelin sheath formation. While in the peripheral nervous system (PNS) myelin is produced by Schwann cells, in the CNS this task is performed by oligodendrocytes that differentiate from OPCs, the main self-renewing population of cells in CNS [22]. OPCs differentiate and mature into myelinating oligodendrocytes through intrinsic and extrinsic regulatory mechanisms. The latter, then, wrap around the axons, expanding their membranes in specific locations, and, therefore, creating a myelin sheath [23]. OPCs are a precursor cell population throughout the CNS representing around 5% to 10% of the total adult brain cells, and are found, together with oligodendrocytes, primarily in white matter regions [24].

OPCs respond to a complex network of molecular cues that orchestrate their differentiation into mature, myelinating oligodendrocytes. Platelet-derived growth factors (PDGFs) [25], fibroblast growth factors (FGFs) [26], and neuronal activity-dependent signals [27] play pivotal roles in this differentiation process. These molecules have also been shown to take on a significant role in restoring myelin, thus unveiling promising therapeutic perspectives [28,29]. Neuregulin (Nrg-1), characterized by a sequence like that of the epidermal growth factor (EGF) [30], acting through ErbB receptor tyrosine kinases (primarily ErbB2/3/4 heterodimers), critically modulates myelin thickness and regenerative responses following injury [31], while moderating astrocyte and microglia reactivity [32]. Concurrently, signaling pathways such as Notch and Wnt/β-catenin precisely regulate OPC maturation timing and OPC proliferation and differentiation, respectively [33,34]. Myelination in the CNS follows a precise temporal and spatial pattern, beginning prenatally and continuing into early adulthood, while white matter myelination during infancy has been found to be linked with spatial gradients and myelin content at birth [35]. Specific transcription factors Olig1, Olig2, MyRF, and Sox10 drive lineage specification and initiate expression of myelin-specific proteins, thereby governing the initiation, extent, and spatiotemporal pattern of CNS myelination [36,37].

The timing of myelination is critical for proper neural circuit formation and function. Typically, sensory pathways myelinate before motor pathways, and regions involved in basic functions myelinate before those associated with higher-order functions [38]. Furthermore, current research reveals a strong relationship between the rhythmic patterns of cortical high-frequency responses and the amount of myelin in specific regions, highlighting how regional myelination contributes to the accurate timing of cortical neuron activities [39] and implicating the extension of these findings to the clinical neurophysiology, particularly the diagnosis of cortical impairments. Moreover, the activity-dependent release of factors such as glutamate and γ-aminobutyric acid (GABA) dynamically regulate OPC proliferation and differentiation [40]. This suggests a dynamic interaction between neurons and oligodendrocytes, where myelination is adjusted based on functional demands.

Axons themselves actively contribute to the regulation of myelination by releasing molecular signals that influence oligodendrocyte behavior. Among these, leucine-rich repeat and immunoglobulin domain-containing Nogo receptor-interacting protein-1 (LINGO-1) act as a key negative regulator of oligodendrocyte differentiation and myelination. Nrg-1 also modulates axon–glia interactions. Contactin-associated protein (Caspr), a critical component of the paranodal junction complex, contributes to the organization of axonal domains and the stabilization of the myelin sheath [41,42]. These molecules mediate essential communication between axons and oligodendrocytes, shaping both the initiation and maintenance of myelin. Disruptions in their expression or signaling balance can result in myelination defects and are implicated in the pathogenesis of various demyelinating diseases. Understanding these axon-derived cues opens avenues for therapeutic strategies aimed at enhancing remyelination and mitigating neurodegenerative processes.

These axon–glia signaling processes are highly susceptible to dysregulation, which underpins various pathological forms of myelin damage, such as demyelination (myelin loss post-development), dysmyelination (abnormal myelin formation), or hypomyelination (insufficient myelin synthesis). Age profoundly influences myelin’s adaptive capabilities. Contrary to earlier assumptions that myelination ceases after adolescence, recent evidence demonstrates that myelin remodeling continues well into adulthood [43]. This persistent myelination plays a vital role in the refinement of neural circuits across the lifespan, highlighting the dynamic and adaptive nature of myelin plasticity [44].

Myelin plasticity includes both the formation of new myelin on previously unmyelinated axons and the remodeling of existing sheaths, such as alterations in internode length, sheath thickness, and node structure [45]. Such dynamic changes in myelin’s architecture and distribution are pivotal for modulating the properties of neuronal networks—they influence the mechanical stability of myelin, as well as its length and thickness, thereby affecting the speed of neural signal transmission and the synchronization of inputs across the nervous system. Moreover, these adjustments in myelin contribute to the precision of neural signaling and can enhance the efficiency of communication within the brain, supporting more complex cognitive functions and adaptive behaviors.

For instance, increased myelin thickness and optimized internode length can lead to faster signal propagation along axons, which is crucial for the rapid processing of information and the execution of complex tasks [46]. The developmental phase of myelination, particularly evident during juvenile growth, sheds light on the principles of myelin plasticity; the established patterns and mechanisms lay the groundwork for ongoing myelination and remyelination processes that continue into adulthood. Such lifelong myelination reflects the brain’s capacity to adapt to new information, experiences, and environmental factors, illustrating the sophisticated and resilient nature of neural circuits [47,48]. This adaptability facilitated by myelin plasticity not only supports learning and memory by refining the efficiency and speed of neural communication but also plays a critical role in the brain’s recovery from injury [49]. The ability to form new myelin sheaths or repair damaged ones is crucial in restoring neural function.

The results of imaging studies have pointed to the capacity of myelin to influence the recovery of cognitive functions in the short and medium term, support neuroregeneration, and increase the white matter volume after practicing demanding tasks [50]. The potential of targeting myelin plasticity in therapeutic strategies and rehabilitation for NDs and brain injuries, especially combined with environmental enrichment and pharmacotherapy, has been largely underscored [51,52]. Additionally, recent research has begun to explore the molecular and cellular mechanisms underlying myelin plasticity, revealing the role of various growth factors, signaling molecules, and transcription factors in regulating myelination and remyelination processes. Regulatory T-cells (T_reg_) have been shown to promote remyelination and oligodendrocyte differentiation [53]. Additionally, the phenotype of neonatal microglia provides necessary signals for myelination. Animal studies have further confirmed the important role of brain-derived neurotrophic factor (BDNF) in promoting myelin repair [54], primarily through its action on Tropomyosin receptor kinase B (TrkB) receptors expressed on both neurons and oligodendrocytes [55]. Collectively, these findings highlight promising therapeutic avenues aimed at enhancing brain function and resilience by modulating myelin plasticity. Such strategies offer new potential for treating neurological disorders and improving cognitive health across the lifespan.

Finally, these findings open the door to the hypothesis that neurodegenerative diseases (NDs) are strongly associated with myelin abnormalities and, by extension, with oligodendrocyte-specific mechanisms such as demyelination, hypomyelination, and dysmyelination [56]. The continuous processes of myelination and remyelination throughout the human lifespan underscore the brain’s remarkable capacity for adaptation and reorganization in response to learning, environmental stimuli, and neurobiological challenges. This dynamic interplay between myelin plasticity and neuroadaptation highlights the central role of oligodendrocytes in maintaining cognitive and functional resilience.

Aging exerts a profound influence on both myelination and remyelination. With advancing age, the efficiency of oligodendrocyte precursor cell (OPC) differentiation and the overall capacity for remyelination decline significantly [57]. This age-related decrease in myelin plasticity contributes to the heightened vulnerability of the aging brain to neurodegenerative disorders [58]. Experimental studies have demonstrated that aged brains exhibit delayed remyelination kinetics and reduced OPC proliferation, likely due to alterations in the cellular microenvironment [59]. Contributing factors include increased pro-inflammatory signaling, changes in extracellular matrix composition, and diminished availability of trophic and growth factors [60]. Understanding these age-dependent shifts in the regulation of myelin dynamics is essential for the development of targeted interventions to enhance remyelination and preserve cognitive function in older individuals.

## 3. Guardians of Neural Integrity: The Critical Role of Oligodendrocytes from Normal Function to Neuroinflammation

As the myelinating glial cells of the CNS, oligodendrocytes are fundamental to neural function, facilitating the rapid conduction of electrical impulses along axons and ensuring axonal health. A critical question concerns the downstream effects of oligodendrocyte damage and the molecular mechanisms that mediate its impact.

Given their role in enabling saltatory conduction, the loss or dysfunction of oligodendrocyte precursor cells (OPCs) and mature oligodendrocytes leads to impaired myelination and subsequent neurological deficits. Demyelination is commonly associated with inflammation or trauma; however, it can also be triggered by genetic factors [61]. Regardless of etiology, myelin disorganization is associated with disrupted expression of key membrane molecules at the paranodal and juxtaparanodal regions, which become exposed following demyelination [62].

Molecules such as Caspr (paranodal), whose axonal levels are downregulated during demyelination [63], and voltage-gated sodium (Nav) channels (juxtaparanodal) [64], serve as molecular indicators of both demyelination and remyelination. In addition to these disruptions, the loss of neurofascin (Nf) and contactin-1 (Cntn-1)—involved in the structural organization of myelin and the stabilization of axon–myelin interactions—further compromises neuronal communication [65,66]. Notably, in several demyelinating disorders including multiple sclerosis (MS) and Guillain–Barré syndrome (GBS), autoantibodies targeting axonal proteins such as Nf155, Cntn-1, and Caspr1 have been detected [67]. One may hypothesize that the exposure of these axonal proteins, following myelin degradation, is a prerequisite for the development of autoimmunity.

Beyond their role in myelination, oligodendrocytes actively participate in the inflammatory responses of the CNS. Neuroinflammation plays a central role in many neurodegenerative diseases, contributing to oligodendrocyte damage and impaired remyelination [68]. Oligodendrocytes and OPCs engage in dynamic crosstalk with microglia and astrocytes, shaping the inflammatory milieu [69]. In conditions such as MS and Alzheimer’s disease (AD), activated microglia release proinflammatory cytokines that exacerbate oligodendrocyte injury and hinder repair processes [70]. Oligodendrocytes themselves can also secrete inflammatory mediators, further influencing disease progression [71]. Understanding the reciprocal relationship between oligodendrocytes and immune signaling is crucial for developing interventions that attenuate neuroinflammation and restore myelin integrity.

Another key function of oligodendrocytes is the regulation of iron homeostasis in the CNS, which is essential for mitochondrial function, neurotransmitter synthesis, and overall neuronal metabolism [72]. Dysregulation of iron handling, including its accumulation in the brain, is a common pathological feature across multiple neurodegenerative diseases [73].

The consequences of myelin dysfunction extend beyond conduction deficits. Myelin is essential not only for insulation but also for protecting axons from extracellular stressors and providing critical metabolic support [74]. What, then, triggers oligodendrocyte dysfunction at the molecular level?

Oligodendrocyte dysfunction and subsequent demyelination are central features of multiple sclerosis (MS) [75], the most prevalent demyelinating disorder of the CNS. MS is characterized by an autoimmune attack against myelin components, leading to inflammation, demyelination, and the formation of sclerotic plaques [76]. Within this autoimmune context, oligodendrocyte malfunction and apoptosis contribute to axonal degeneration and progressive neurological decline [77]. The targets of the immune response differ across disease phases [78], and the involvement of both CD4⁺ and CD8⁺ T cells, as well as B cells, contributes to a complex immunopathology. Autoantibodies against myelin components such as myelin oligodendrocyte glycoprotein (MOG) and myelin basic protein (MBP) have been identified [78]. Genetic studies have linked MS susceptibility to mutations in human leukocyte antigen (HLA) genes [79], and epigenetic factors—such as methylation of the interleukin-2 receptor alpha gene (IL2RA, SNP rs2104286), which modulates CD8^+^ T cell function—have also been implicated [80].

The diversity of immune, genetic, and environmental factors involved in demyelinating diseases is further exemplified by related conditions. Neuromyelitis optica spectrum disorders (NMOSDs), also known as Devic’s disease, primarily affect the optic nerves and spinal cord and are characterized by autoantibodies against aquaporin-4 water channels on astrocytes [81]. Acute disseminated encephalomyelitis (ADEM) is a post-infectious inflammatory demyelination of the brain and spinal cord with multifocal lesions [82]. Progressive multifocal leukoencephalopathy (PML) is caused by JC virus reactivation in immunosuppressed individuals, leading to widespread white matter destruction [83].

Peripheral nervous system (PNS) demyelinating diseases include GBS—an acute inflammatory disorder often triggered by infection, leading to rapid-onset weakness and paralysis—and chronic inflammatory demyelinating polyneuropathy (CIDP), its chronic counterpart. Charcot–Marie–Tooth (CMT) disease represents a group of inherited demyelinating neuropathies marked by mutations in genes involved in myelin maintenance, resulting in progressive muscle atrophy and sensory deficits [84].

The pathophysiology of myelin disorders involves a complex interplay between immune-mediated processes, genetic predispositions, and environmental triggers. In autoimmune diseases such as MS, myelin-specific immune responses lead to inflammation and lesion formation [85]. In genetic disorders like CMT, mutations impair the synthesis or function of essential myelin proteins [86]. In metabolic disorders, enzymatic deficiencies compromise myelin integrity and turnover [87,88]. While the origins of myelin damage vary widely, it is ultimately the structural integrity of the myelin sheath that determines axonal survival. Section 4 explores how disruptions in myelin itself—via developmental failure or acquired degeneration—give rise to diverse clinical entities.

## 4. When Protection Fails: Exploring Disorders of Myelin Loss and Dysfunction

Myelin disorders, including leukodystrophies and acquired demyelinating diseases, encompass a diverse group of neurological conditions characterized by the disruption, insufficient formation, or degeneration of the myelin sheath. Although traditionally categorized by etiology—genetic, autoimmune, metabolic, or environmental—growing evidence suggests that distinctions between dysmyelination, hypomyelination, and demyelination reflect overlapping mechanistic continua rather than isolated categories [89]. Rare myelin disorders, while individually uncommon, offer unique insights into shared cellular vulnerabilities and have been instrumental in advancing our understanding of oligodendrocyte pathophysiology.

One such example is hypomyelination with atrophy of the basal ganglia and cerebellum (H-ABC), a rare leukodystrophy characterized by regionally selective and progressive white matter failure. First defined by a distinctive MRI pattern, this condition illustrates how structural brain development is tightly coupled to spatially regulated myelin production, and how regional susceptibility to hypomyelination can shape clinical outcomes [90].

Another important group is the GM2 gangliosidoses, including Tay–Sachs and Sandhoff syndrome, which exemplify how metabolic defects contribute to myelin disruption. These lysosomal storage disorders are caused by deficiencies in β-hexosaminidase, resulting in the accumulation of gangliosides that perturb lipid processing essential for myelin biosynthesis. In a murine model of Sandhoff disease, gene therapy delivered in the early symptomatic phase led to marked clearance of stored glycolipids and neuroinflammatory markers, extending survival and improving neurological function [91]. However, myelination defects—once established—remained largely irreversible, highlighting a narrow therapeutic window in which functional neurorecovery and oligodendrocyte plasticity may be possible. These findings underscore that early intervention is critical in disorders where developmental myelination is at risk and reinforce the notion that myelin plasticity is temporally constrained.

PMD represents a prototypical X-linked leukodystrophy caused by mutations in the proteolipid protein 1 (PLP1) gene. Variations in PLP1 copy number—including duplications, deletions, and point mutations—lead to abnormal myelin compaction and oligodendrocyte dysfunction [92]. Notably, experimental work has demonstrated that neural stem cell transplantation can promote remyelination and partially restore myelin architecture in animal models of PMD [93]. Interestingly, PLP1 mutations have also been linked to multiple sclerosis (MS), where they appear to facilitate pathogenic immune responses and lymphocyte activation, suggesting that genetic and inflammatory mechanisms may converge on common oligodendroglial targets [94].

A different perspective is offered by Cockayne syndrome (CS), a disorder of impaired DNA repair that also results in prominent white matter pathology. Neuropathological and imaging studies have described tigroid leukodystrophy, reflecting regional oligodendrocyte damage and abnormal myelin distribution [95]. While some studies suggest delayed and region-specific myelination, others debate whether myelin loss stems from underproduction or secondary degradation [96,97]. CS thus highlights how systemic deficits—in this case, DNA repair and vascular integrity—can selectively impair oligodendrocyte function, even outside traditional immune or metabolic frameworks.

Expanding on the understanding of GM2 disorders, recent clinical and molecular analyses of Tay–Sachs disease have reaffirmed the presence of significant early white matter involvement alongside neuronal degeneration. Far from being confined to gray matter pathology, Tay–Sachs now represents a broader model of combined storage, inflammatory, and myelin-related pathology, underscoring the importance of integrated glial–neuronal dynamics in disease progression [98].

Taken together, these rare disorders reveal convergent mechanisms by which diverse triggers—genetic mutations, lysosomal dysfunction, DNA repair deficits—impair oligodendrocyte maturation, stability, and function. Despite differences in onset, pathology, and progression, they all underscore the central role of oligodendrocytes as vulnerable integrators of neuronal and systemic signals. Crucially, they demonstrate that myelin disruption may precede—or potentiate—neurodegeneration, inviting a reconsideration of white matter’s role in neurological disease.

## 5. Beyond Neurons: The Impact of Myelin Dysfunction in Other Neurodegenerative Disorders

**Figure 1:**
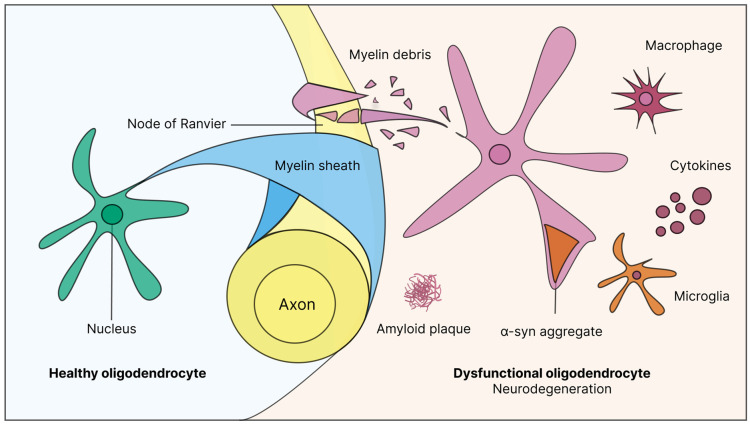
Oligodendrocyte function in healthy vs. diseased states.

Oligodendrocytes, long studied in the context of classical myelin disorders, are now recognized as key players in broader NDs, including AD, PD, and ALS/MND [99]. Recent advancements in neuroscientific research now unravel not only the complex interactions between OPCs, oligodendrocytes, and neurons, but also adaptive myelination in memory and learning, and myelin plasticity, shedding light on how disruptions in these interactions contribute to the pathology of NDs [15]. These findings underscore the importance of understanding oligodendrocyte biology, not solely for understanding ND pathogenesis, but to explore novel therapeutic avenues aimed at protecting, repairing, or replacing damaged oligodendrocytes. Studies have illuminated significant roles for oligodendrocyte dysfunction and demyelination in the neuropathology of AD, PD, and ALS/MND (Figure 1). Myelin alterations may actively contribute to disease progression rather than merely representing secondary consequences [100,101]. The following sections detail the specific contributions of oligodendrocyte dysfunction in AD, PD, and ALS.

Alzheimer’s disease (AD) has traditionally been studied through the lens of neuronal pathology, yet mounting evidence implicates oligodendrocytes in its progression. In AD, myelin breakdown and oligodendrocyte loss are observed in post-mortem brains, particularly in regions susceptible to AD pathology, such as the hippocampus and cortex [102]. This cell loss is associated with cognitive decline and disease progression, suggesting that oligodendrocyte dysfunctions may contribute to the neurodegenerative process in AD. The accumulation of amyloid-beta (Aβ) plaques, a hallmark of AD, has been shown to damage oligodendrocytes and disrupt myelin integrity [103], exerting toxic effects on oligodendrocytes. Aβ oligomers have been shown to induce apoptosis in oligodendrocyte cultures, and post-mortem studies have revealed Aβ accumulation within oligodendrocytes in AD brains, directly affecting oligodendrocyte survival and function [104]. AD is characterized by significant myelin breakdown, driven by the ε4 allele of a lipid transporter apolipoprotein E (APOE4), which contributes to the disruption of neuronal communication and cognitive deficits [105]. The loss of myelin integrity in AD may be due to both the direct effects of Aβ on oligodendrocytes and the failure of these cells to adequately maintain and repair myelin sheaths [106].

Furthermore, myelin alterations in AD may exacerbate neuronal dysfunction by impairing signal transmission and contributing to tau pathology, another key feature of AD [107]. Magnetic resonance imaging (MRI) studies in AD patients have consistently found white matter hyperintensities and structural abnormalities, reflecting myelin loss and axonal damage [108]. Diffusion tensor imaging has further elucidated these changes, showing altered white matter integrity and connectivity in AD, which correlates with disease severity and cognitive impairment [109]. Additionally, in response to myelin damage, OPCs are mobilized for repair and remyelination processes. However, in AD, this response appears to be impaired, with studies indicating a reduced capacity for OPCs to differentiate into mature oligodendrocytes and remyelinate axons effectively, as OPCs are particularly vulnerable to hypoxia-ischemia, neuroinflammation, as well as amyloid deposition [110]. This impairment may contribute to the progressive myelin degradation observed in the disease. Tau pathology has also been observed in oligodendrocytes in certain tauopathies and some AD models [111]. Abnormal hyperphosphorylated tau can accumulate in oligodendrocytes, potentially disrupting their function and contributing to myelin abnormalities. The crosstalk between tau pathology and oligodendrocyte dysfunction is an area of active research [112]. The emerging understanding of oligodendrocyte involvement in AD suggests new therapeutic targets. Strategies aimed at protecting oligodendrocytes from Aβ toxicity, enhancing OPC differentiation and myelin repair, and addressing tau pathology within oligodendrocytes may offer novel approaches to slowing disease progression and improving cognitive function in AD [106,113].

Oligodendrocytes also play a significant role in PD, a ND primarily characterized by the degeneration of dopaminergic neurons in the substantia nigra pars compacta and the presence of Lewy bodies composed of α-synuclein (α-syn) [114]. Recent research has begun to unravel the complexities of oligodendrocyte involvement in PD, providing new insights into its pathology and potential therapeutic targets. In PD, α-syn not only accumulates in neurons but also in the oligodendrocytes, forming glial cytoplasmic inclusions (GCIs) [115]. These inclusions are a hallmark of multiple system atrophy (MSA), a Parkinsonism disorder, and are also observed in classical PD cases [116]. The accumulation of α-syn in oligodendrocytes is toxic and contributes to myelin dysfunction and oligodendrocyte death [117]. PD is associated with demyelination and white matter abnormalities, as evidenced by neuroimaging and post-mortem studies [118]. These changes contribute to the disruption of neuronal circuits and are linked with motor and cognitive symptoms observed in PD [119]. The exact mechanisms by which α-syn pathology leads to myelin loss are still under investigation but may involve direct toxicity to oligodendrocytes and interference with their function.

Similar to AD, PD affects the dynamics of OPCs. There is evidence to suggest that the disease process in PD may alter the proliferation, migration, and differentiation of OPCs, impacting the brain’s capacity for myelin repair and maintenance [120]. Oligodendrocytes are particularly susceptible to mitochondrial dysfunction and oxidative stress, both of which are prominent features of PD neuropathology. The high metabolic demand of producing and maintaining myelin makes oligodendrocytes vulnerable to energy deficits and oxidative damage, potentially exacerbating myelin loss and neuronal dysfunction in PD [15]. Some studies have reported mercury presence in neurons and oligodendrocytes in PD-affected brain regions, frequently in conjunction with accumulated α-syn aggregates. The presence of mercury in the motor cortex, thalamus, and striatum is associated with symptoms such as bradykinesia and rigidity, whereas its accumulation in the cerebellum could be linked to the manifestation of tremors [121]. However, the significance of this finding remains debated and requires further validation. Neuroinflammatory responses in PD also impact oligodendrocytes. Microglia activation and the release of pro-inflammatory cytokines can contribute to oligodendrocyte stress and death, further compromising myelin integrity [122]. Strategies aiming to reduce α-syn accumulation in oligodendrocytes protect oligodendrocytes from mitochondrial dysfunction and oxidative stress, enhance OPC function for effective myelin repair, and modulate neuroinflammatory responses could provide novel approaches for treating PD.

The involvement of oligodendrocytes in ALS/MND, a devastating ND characterized by the progressive loss of motor neurons in the spinal cord, brain stem, and motor cortex, highlights the multifaceted nature of the disease [123]. Recent scientific insights have begun to elucidate the critical roles that oligodendrocytes and myelin dysfunction play in ALS/MND, suggesting mechanisms that contribute to motor neuron degeneration and potential therapeutic targets [124]. Research has identified oligodendrocyte degeneration as a feature of ALS/MND neuropathology [123]. Loss of oligodendrocytes in the vicinity of degenerating motor neurons suggests a supportive role of these glial cells in motor neuron health and function, exceeding the axonal pathology in white matter [125]. The degeneration of oligodendrocytes may precede or accompany motor neuron loss, implying a contributory role to disease progression [126]. As highlighted, oligodendrocytes provide metabolic support to axons, including lactate as an energy source through the monocarboxylate transporter [127]. In ALS/MND, this supportive function is compromised, potentially leading to axonal dysfunction and degeneration [124]. The disruption in metabolic support from oligodendrocytes to motor neurons is a key area of interest in understanding ALS/MND. Furthermore, the disease process in ALS/MND impairs the ability of OPCs to mature and remyelinate affected axons effectively, exacerbating motor neuron vulnerability [128]. Oligodendrocytes are involved in glutamate metabolism, acting to clear this neurotransmitter from synaptic spaces [129]. In ALS/MND, dysregulation of glutamate metabolism by oligodendrocytes can contribute to excitotoxicity, a condition where excessive glutamate stimulation leads to neuronal damage and death [130]. Finally, ALS/MND features a neuroinflammatory component where activated microglia and astrocytes release pro-inflammatory cytokines [131]. These cytokines can further stress oligodendrocytes and OPCs, hindering their survival and function. The inflammatory milieu in ALS/MND thus indirectly affects motor neuron health through oligodendrocyte dysfunction [132]. The growing understanding of oligodendrocyte involvement in ALS/MND suggests several therapeutic strategies, such as enhancing oligodendrocyte survival by protecting them from degeneration; promoting remyelination through therapies aimed at stimulating OPC differentiation and remyelination; regulating glutamate metabolism through the support of oligodendrocyte function to reduce excitotoxicity and protect motor neurons; and anti-inflammatory therapies.

Emerging evidence suggests that oligodendrocytes are also implicated in other neurodegenerative diseases, including Huntington’s disease (HD) [133,134,135]. Research indicates that there are significant alterations in the white matter and myelin integrity in individuals with HD, suggesting that myelin breakdown may play a role in disease progression [136,137].

Beyond the major neurodegenerative diseases, additional evidence implicates oligodendrocytes in several other conditions characterized by white matter disruption, such as essential tremor and frontotemporal dementia. Essential tremor, characterized by involuntary rhythmic shaking, has been associated with white matter changes and oligodendrocyte dysfunction [138]. In frontotemporal dementia, oligodendrocyte loss and myelin damage are observed, contributing to the progressive cognitive decline and behavioral changes seen in patients [139]. These findings further underscore the critical involvement of oligodendrocytes in a broad spectrum of neurodegenerative conditions.

## 6. Therapeutic Perspectives

Therapeutic efforts targeting myelin repair and oligodendrocyte function are gaining momentum in the context of neurodegenerative disease management. Promising avenues span pharmacological agents, (epi) genetic interventions, and cell-based regenerative strategies, particularly those involving induced pluripotent stem cells (iPSCs).

Pharmaceutical compounds with remyelinating potential are currently under intense investigation. Among these, clemastine fumarate, a first-generation antihistamine, has demonstrated efficacy in enhancing OPC differentiation and promoting myelin repair in multiple sclerosis (MS) models and early clinical trials [140,141]. Other compounds, such as metformin and high-dose biotin, are being evaluated for their neuroprotective and remyelinating effects in demyelinating conditions, with preliminary findings suggesting improved metabolic support and oligodendrocyte survival [142,143].

Beyond small molecules, epigenetic therapies represent a frontier in personalized intervention. Technologies such as CRISPR/Cas9 offer the potential to correct specific genetic mutations underlying myelin disorders [144], while modulation of DNA methylation and histone acetylation pathways has been shown to influence OPC differentiation and remyelination capacity [145,146]. By targeting these epigenetic signatures, researchers aim to reactivate endogenous repair programs and restore functional myelin in neurodegenerative contexts.

The use of iPSCs offers a transformative approach to regenerative medicine. These reprogrammed cells can be differentiated into OPCs and mature oligodendrocytes, providing an autologous source for cell replacement therapy [147]. Preclinical studies have shown that transplantation of iPSC-derived oligodendrocytes supports remyelination and axonal protection in animal models of demyelination [148]. Importantly, iPSC-based therapies offer the advantage of patient-specific compatibility, reducing the risk of immune rejection while opening avenues for precision neurorepair.

Together, these therapeutic strategies highlight a growing toolkit for targeting myelin restoration and oligodendrocyte resilience in neurodegenerative disorders. Future success will depend on the integration of mechanistic insights with translational frameworks, ensuring both efficacy and safety across diverse clinical applications.

## 7. Conclusions

The present review systematically delineates the pivotal roles of oligodendrocytes and OPCs in the pathophysiology of prominent NDs, including AD, PD, and ALS/MND. By integrating recent scientific advancements, we have emphasized the substantial impact of oligodendrocyte dysfunction, demyelination, and compromised remyelination processes on the progression of neurodegeneration. Our discussion of the cellular and molecular intricacies of oligodendrocytes within the CNS underscores the importance of myelin integrity for neuronal functionality and the severe repercussions of its perturbation.

The associations drawn between the accumulation of neurotoxic proteins—such as Aβ in AD and α-syn in PD—and the potential contributions of environmental toxicants substantiate the interconnectedness of oligodendrocyte health with the nervous system’s vulnerability to degenerative pathways. Furthermore, the discourse on myelin plasticity reveals its vital role in modulating neural circuits—not only during development, but across the lifespan. This dynamism presents an optimistic prospect for therapeutic strategies aimed at promoting remyelination and neuroprotection, underscoring the adaptive capacity of OPCs to meet evolving neuronal demands.

Understanding the regulatory pathways that govern OPC differentiation—such as Wnt, Notch, and epigenetic networks—will be essential for translating these insights into effective molecular therapies. Moreover, future therapeutic design must embrace a personalized approach, accounting for interindividual variability in glial responses, genetic susceptibility, and disease trajectory.

Despite these insights, many aspects of oligodendrocyte regulation, myelin pathology, and their links to neurodegenerative phenomena remain to be elucidated. The prevalence and profound societal impact of NDs underscore the urgency for expanded research into these mechanisms. Advancing our understanding of oligodendrocyte contributions to neurodegeneration represents a formidable challenge, yet one that holds promise for groundbreaking therapeutic avenues. Through concerted research efforts, cross-disciplinary collaboration, and innovative methodologies, we can begin to unravel the complexities of these disorders—paving the way toward alleviating their devastating clinical and societal burdens.

## Figures and Tables

**Table 1:** Summary of oligodendrocyte and OPC dysfunction, clinical symptoms, and potential therapeutic targets across major neurodegenerative diseases.

Disease	Main Symptoms	Oligodendrocyte Dysfunction	Potential Therapeutic Targets
Alzheimer’s Disease (AD)	Cognitive decline, memory loss	Myelin breakdown, oligodendrocyte loss, impaired OPC differentiation	Enhance remyelination, target tau hyperphosphorylation, support OPC viability
Parkinson’s Disease (PD)	Motor dysfunction, cognitive impairment	Demyelination, α-synuclein accumulation, mitochondrial stress in oligodendrocytes	Reduce α-synuclein burden, enhance oxidative stress resistance
Amyotrophic Lateral Sclerosis/Motor Neuron Disease (ALS/MND)	Progressive muscle weakness, paralysis	Metabolic support failure, glutamate excitotoxicity, impaired OPC maturation	Support oligodendrocyte survival, regulate glutamate metabolism, promote OPC differentiation
Multiple Sclerosis (MS)	Motor, sensory, and cognitive deficits	Immune-mediated demyelination, OPC maturation arrest, chronic inflammation	Stimulate remyelination, modulate neuroinflammation, promote OPC maturation
